# Paclitaxel coated balloon versus conventional balloon angioplasty in dysfunctional dialysis arteriovenous fistula: a systematic review and meta-analysis of randomized controlled trials

**DOI:** 10.1080/0886022X.2022.2029487

**Published:** 2022-02-15

**Authors:** Chuxuan Luo, Mingzhu Liang, Yueming Liu, Danna Zheng, Qiang He, Juan Jin

**Affiliations:** a Division of Health Sciences, Hangzhou Normal University, Hangzhou, Zhejiang, China; b Department of Nephrology, Zhejiang Provincial People’s Hospital, Affiliated People's Hospital, Hangzhou Medical College, Hangzhou, Zhejiang, China; c The Medical College of Qingdao University, Qingdao, Shandong, China; d Department of Nephrology, the First People's Hospital of Hangzhou Lin'an District, Affiliated Lin'an People's Hospital, Hangzhou Medical College, Hangzhou, Zhejiang, China

**Keywords:** Paclitaxel, angioplasty, balloon, arteriovenous fistula, meta-analysis

## Abstract

**Purpose:**

To compare the efficacy and safety between paclitaxel coated balloon (PCB) angioplasty and conventional balloon (CB) angioplasty in the treatment of dysfunctional arteriovenous fistula (AVF).

**Methods:**

We searched four major electronic databases (PubMed, EMBASE, Web of Science and the Cochrane Library) for randomized controlled trials (RCTs) published from inception through November 28, 2021. Outcomes of interest included target lesion primary patency (TLPP), technical success and all-cause mortality. The STATA package version 15.1 was utilized to undertake meta-analyses.

**Results:**

Fourteen RCTs totaling 1535 patients were analyzed. The available data showed that there were no significant differences of TLPP rates at 3, 6, 9 and 12 months between the PCB group and the CB group (risk ratio (RR) 1.00, 95% confidence interval (CI) 0.93–1.07, *p* = 1.000, *I*^2^ = 33.5%, Cochrane *Q* test *p* = 0.185, fixed-effect model; RR 1.17, 95% CI 0.99–1.39, *p* = 0.065, *I*^2^ = 75.4%, Cochrane *Q* test *p* = 0.000, random-effect model; RR 0.81, 95% CI 0.35–1.89, *p* = 0.625, *I*^2^ = 62.8%, Cochrane *Q* test *p* = 0.045, random-effect model; RR 1.19, 95% CI 0.97–1.47, *p* = 0.096, *I*^2^ = 40.5%, Cochrane *Q* test *p* = 0.071, random-effect model). In addition, two groups had similar technical success rates (RR 1.00, 95% CI 0.97–1.03, *p* = 1.000, *I*^2^ = 0.0%, Cochrane *Q* test *p* = 0.596, fixed-effect model) and all-cause mortality rates (RR 1.00, 95% CI 0.54–1.84, *p* = 1.000, *I*^2^ = 0.0%, Cochrane *Q* test *p* = 0.599, fixed-effect model).

**Conclusions:**

PCB angioplasty did not appear to convey any obvious advantage over CB angioplasty in the treatment of dysfunctional AVF. However, further multi-center, large-scale and well-designed RCTs are needed to prove outcomes.

## Introduction

Reliable vascular access is known as the lifeline of maintenance hemodialysis patients. There are several commonly used permanent hemodialysis vascular access types such as autologous arteriovenous fistula (AVF), tunnel-cuffed catheter (TCC) and arteriovenous graft (AVG). However, the extensive application of AVG in clinical practice has not yet been realized due to its high price and technological problems. Note that TCC was chosen only when AVF could not be established or patients were expected to have a relatively short survival time. It has been found that the patients with AVF had a better survival rate compared with patients with other access types [1]. As a consequence, AVF is currently the preferred choice for vascular access. And its functional status directly affects the dialysis efficiency and quality of life of patients undergoing maintenance hemodialysis. Nevertheless, the persistence of AVF was not satisfactory enough and the most prevalent causes of dysfunctional AVF were thrombosis and vascular stenosis [2]. Therefore, long-term patency preservation of the fistula tract presented an urgent clinical problem to be solved. In the past era, dysfunctional AVF was generally treated with surgical methods. With the rapid advancement of intraluminal interventional techniques, it has emerged as a primary therapeutic approach in the treatment of this disease. Conventional balloon (CB) angioplasty was thought to be the gold standard for the treatment of dysfunctional dialysis access, either AVF or AVG. But Haskal’s study showed that the incidence of patency of the treatment area and the access circuit in the CB group was only 23% and 20%, respectively [3]. Compared with CB, high-pressure balloons and cutting balloons are able to improve the patients’ prognosis, but the stenosis rate is still high in the short term [4,5]. Accordingly, the emergence of paclitaxel coated balloon (PCB) is expected to be useful for solving the foregoing issues. The role of PCB in coronary artery diseases and peripheral arterial diseases has been widely recognized [6,7]. However, whether PCB angioplasty outperforms CB angioplasty in the treatment of dysfunctional AVF is still in controversy.

Several studies confirmed a benefit of PCB angioplasty [8–16] while the others showed they were equivalent in target lesion primary patency (TLPP) [17–20]. Moreover, the results of a randomized controlled trial (RCT) showed that the TLPP after PCB angioplasty was even worse [21]. As the safety and benefits of PCB angioplasty remain unknown, we aimed to conduct a meta-analysis to reevaluate the results.

## Materials and methods

The present meta-analysis was reported referring to the Preferred Reporting Items for Systematic reviews and Meta-Analysis (PRISMA) statement [22].

### Search strategy

A systematic search of relevant literature available on PubMed, EMBASE, Web of Science and the Cochrane Library containing several keywords “arteriovenous fistula,” “dialysis fistula,” “drug-coated balloon,” “drug-eluting balloon” and “paclitaxel” published from their date of inception to November 28, 2021, was carried out (Appendix 1). We did not use any language or data restrictions, although we used only English search terms. References of these articles were also searched to find potential relevant articles.

### Inclusion and exclusion criteria

Titles, abstracts and the full texts of all retrieved studies were preliminarily filtrated by a pair of authors to determine the inclusion (LC and LM). Disagreements from the two authors were solved by consensus or by appeal to a third review author (JJ). Inclusion criteria: (1) RCTs with two parallel arms; (2) Hemodialysis patients with dysfunctional AVFs; (3) Patients were treated with PCB angioplasties or CB angioplasties; (4) TLPP rates, technical success rates or all-cause mortality rates of both methods were provided in the literature; (5) Clinical follow-up of at least 6 months. Exclusion criteria: (1) Observational studies, animal studies, *in vitro* tests, reviews, comments, editorials, case reports and series, protocols, letters, conference abstracts, crossover trials and single-arm tests; (2) Repeated reporting; (3) Full text not available; (4) AVF and AVG data reported together; (5) Use of a stent.

### Outcomes of interest and data extraction

The endpoint events were defined in accordance with the Society of Interventional Radiology (SIR) criteria for percutaneous interventional procedures in dialysis access [23] and the previous literature [12,15,19]. TLPP was adjudicated as freedom from clinically-driven target lesion revascularization (CD-TLR) or access circuit thrombosis during the follow-up period. TLPP ended when any one of the followings occurred: (1) decreased access blood flow (<500mL/min, 25% decrease in flow); (2) elevated venous pressures; (3) decreased dialysis dose (Kt/V); (4) abnormal physical exam included: i. diminished or abnormal thrill (focal, systolic only, etc); ii. pulsatility; iii. flaccid access; iv. abnormal bruit; v. arm or hand swelling; (5) prolonged bleeding; (6) difficult puncture; (7) infiltration; (8) recirculation; (9) pulling clots. Technical success was defined as successful completion of the angioplasty procedure with <30% residual stenosis by visual estimate and a palpable thrill. All-cause mortality was reported through 12 months. Data were separately extracted by two review authors (LC and LM).

### Risk of bias and quality assessment

Methodological quality appraisal was conducted by two independent reviewers (LC and LM). In the event of a discrepancy between the two authors, a third author will decide (JJ). The quality evaluation of selected studies was performed using 7 elements from the recommended Cochrane Collaborations tool: random sequence generation (selection bias), allocation concealment (selection bias), blinding of participants and personnel (performance bias), blinding of outcome assessment (detection bias), incomplete outcome data (attrition bias), selective reporting (reporting bias) and other bias [24]. Publication bias was assessed through visual inspection of funnel charts, whereby an asymmetric funnel diagram indicated the presence of publication bias.

### Statistical analysis

Statistical heterogeneity was assessed *via* the *I*^2^ statistic and Cochrane’s *Q* test. If evident heterogeneity (*I*^2^ > 50% or *p* < 0.1), the random-effect model was employed for analysis; if not, the fixed-effect model. We conducted sensitivity analyses and subgroup analyses to search for the potential sources of heterogeneity. A value of *p* < 0.05 was accepted as a statistically significant difference. Meta-analyses were processed with STATA software version 15.1 (StataCorp, College Station, TX, USA) to calculate risk ratios (RRs) and their 95% confidence intervals (CIs). The kappa coefficients were computed by use of SPSS 25.0 (SPSS Inc., Chicago, IL, USA) to assess the degree of concordance between the two investigators.

## Results

**Figure 1.**
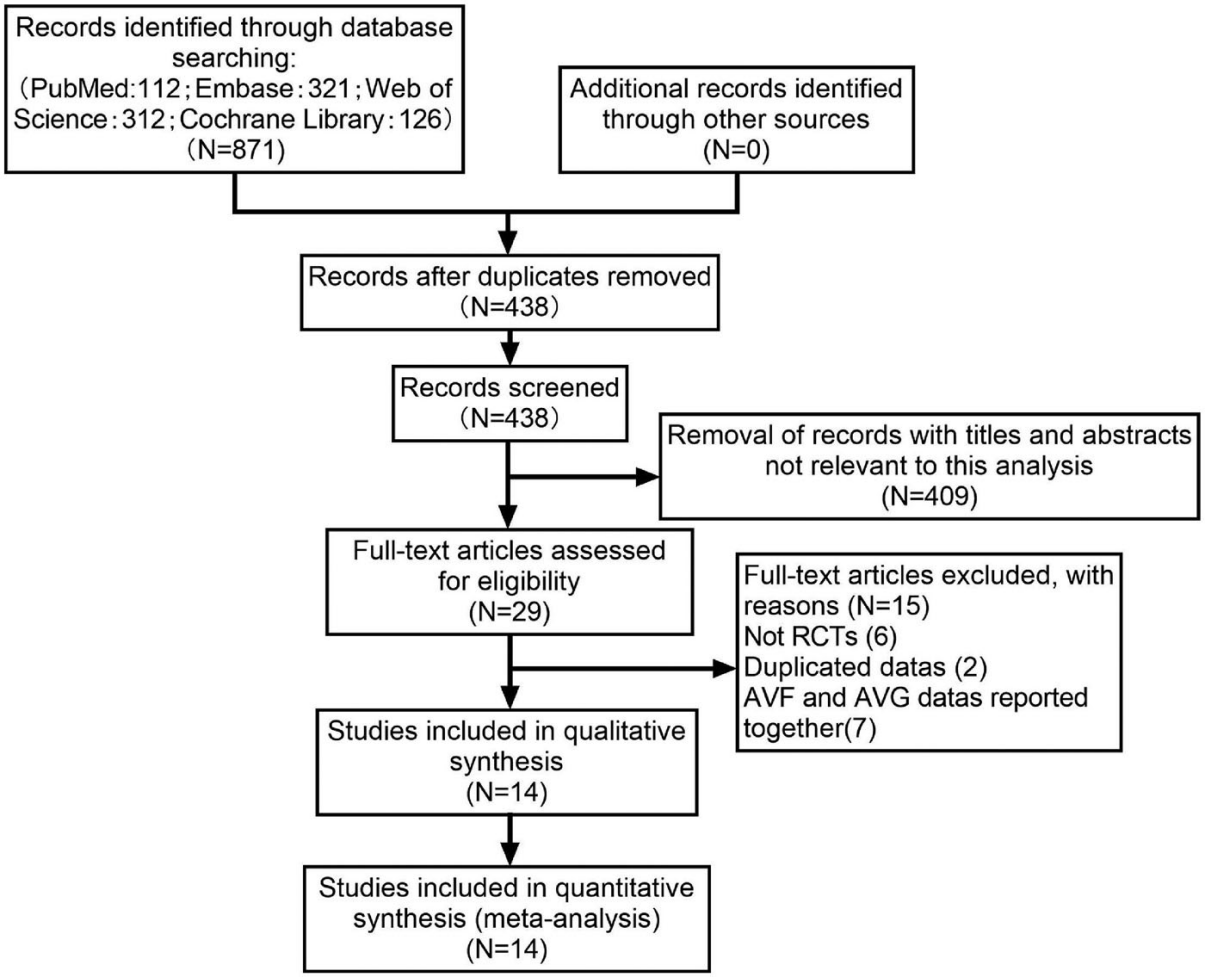
Study selection flow diagram.

**Figure 2.**
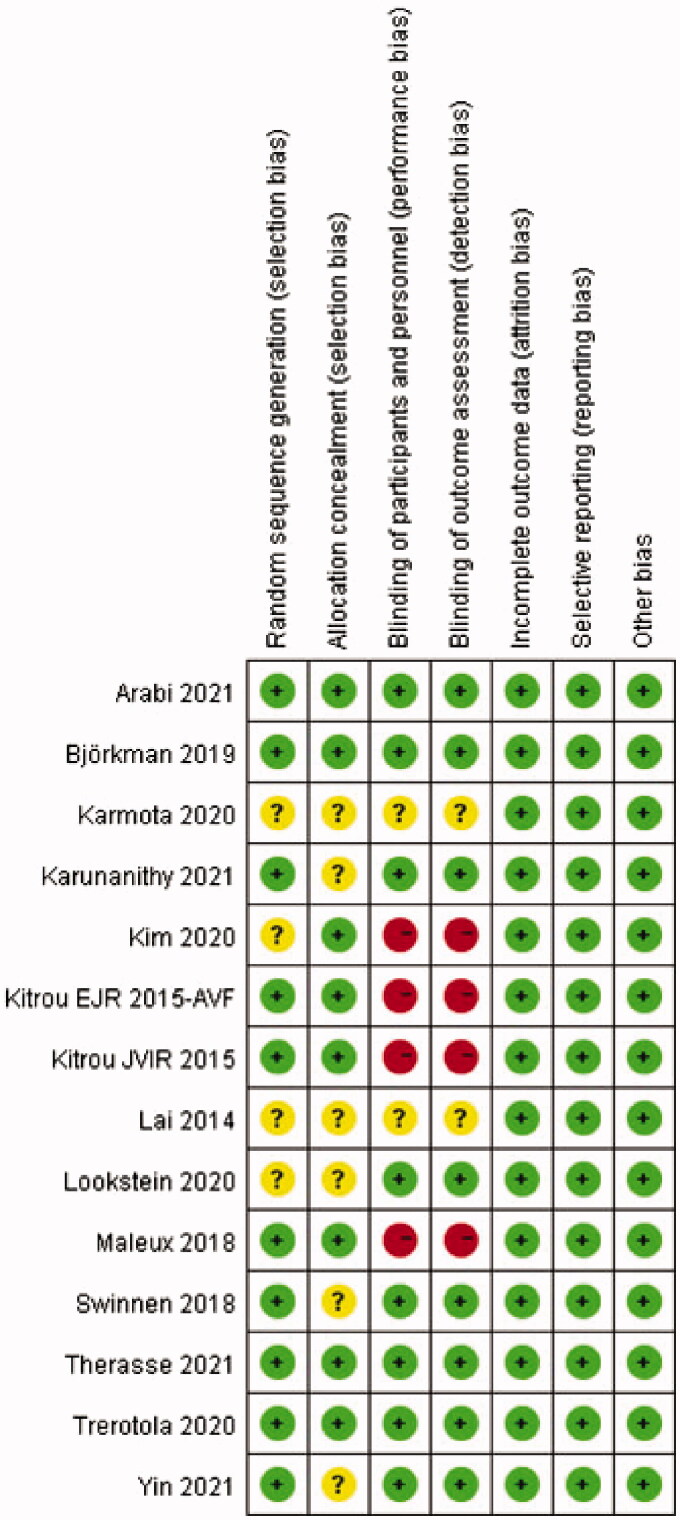
Risk of bias summary in included studies.

**Figure 3.**
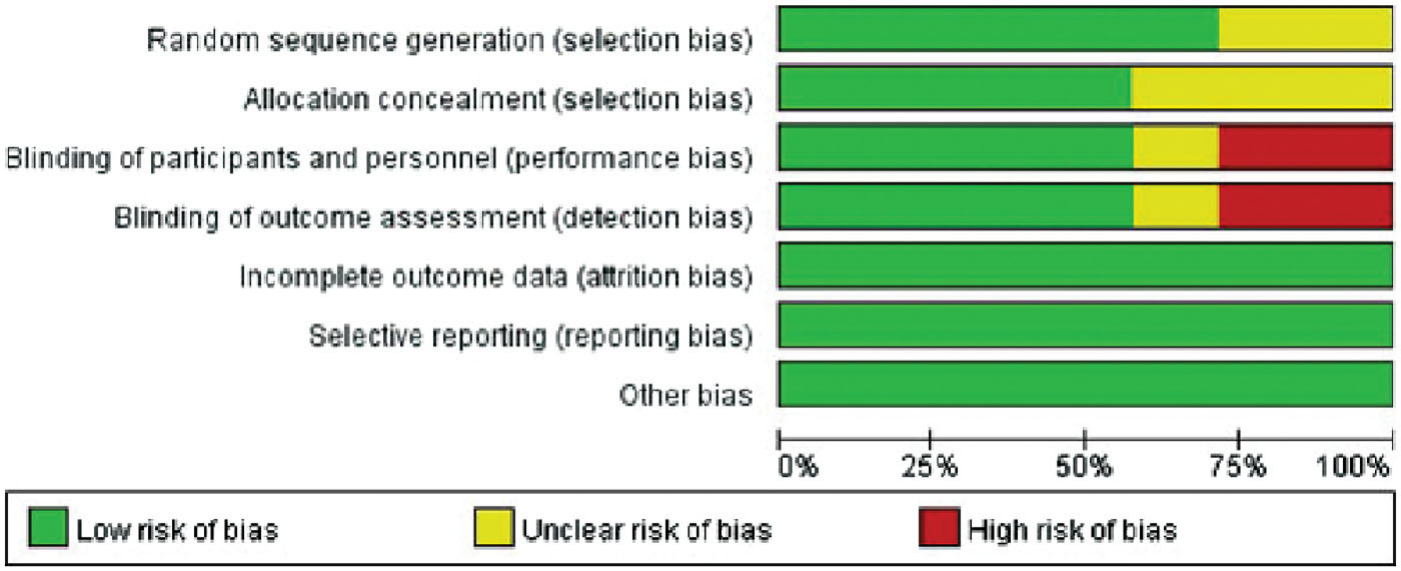
Risk of bias graph in included studies.

We identified 871 potentially eligible studies. After deduplication, 438 documents remained. Of these abstracts, 409 were excluded based upon the inclusion and exclusion criteria. Then, a total of 29 potentially relevant full-texts were retrieved and subjected to further review. Studies excluded and reasons for exclusion after full-text screening were provided in Table 1. Eventually, fourteen RCTs [8–21], including eight multi-center trials [8,12–16,18,20], with 1535 patients fulfilled the criteria for inclusion. A moderately high level of agreement between two independent investigators was observed at the title and abstract review (kappa = 0.695) and full-text evaluation (kappa = 0.861) stages. The detailed steps of the study search and selection process were outlined in Figure 1 and the baseline characteristics of the selected trials were summarized in Table 2. Two of the fourteen studies included patients with AVF and AVG, but we extracted data only for patients with AVF [9,14]. As for the methodological quality assessment, all studies scored three to seven points, among which four articles scored seven points [14,15,17,21]. The kappa values of agreement during the quality appraisal and data extraction were 0.863 and 1.000, respectively. The detailed score of each item for each article was described in Figure 2 and the proportion of each item in the methodological evaluation was shown in Figure 3.

**Table 1.** List of records excluded after full-text reading.

Author, year	Reason for exclusion
Ali 2020 [25]	1
Bjorkman 2021 [26]	2
Eldmarany 2020 [27]	3
Irani 2018 [28]	1
Karnabatidis 2021 [29]	3
Katsanos 2012 [30]	1
Moreno-Sánchez 2019 [31]	1
Pang 2021 [32]	1
Patanè 2019 [33]	3
Rai 2019 [34]	3
Roosen 2017 [35]	1
Teo 2013 [36]	1
Trerotola 2018 [37]	2
Verbeeck 2016 [38]	3
Yildiz 2019 [39]	3

1: arteriovenous fistula and graft datas reported together; 2: duplicated datas; 3: not randomized controlled trials.

**Table 2.** Baseline characteristics of included studies.

Author, year	Cases		Male%		Age		Diabetes mellitus%		Hypertension%		Device type	Inflation time
	PCB	CB	PCB	CB	PCB	CB	PCB	CB	PCB	CB		
Arabi, 2021 [17]	12	11	41.7	54.5	68.7 ± 10.1	66.8 ± 13.7	91.7	72.7	100	90.9	PCB: Lutonix 035, Bard (2 µg/mm^2^) Control: unspecified	120 sec
Björkman, 2019 [21]	21	18	55.6	72.2	67.4 (46–87)	67.0 (28–82)	61.1	61.1	88.9	77.8	PCB: IN. PACT Admiral, Medtronic (3.5 µg/mm^2^) Control: unspecified	90 sec
Karmota, 2020 [8]	30	30	43.3	53.3	54.7 ± 13.2	49.2 ± 11.5	63.3	50.0	50.0	56.7	PCB: Lutonix 035, Bard (dose unspecified) Control: unspecified	180 sec
Karunanithy, 2021 [18]	106	106	63.2	57.5	66.9 ± 12.7	64.1 ± 13.3	54.7	43.4	–	–	PCB: Lutonix 035, Bard (2 µg/mm^2^) Control: Ultraverse, Bard	Unspecified
Kim, 2020 [19]	20	19	60.0	47.4	60.7 ± 12.2	63.7 ± 11.8	80.0	78.9	–	–	PCB: IN. PACT Admiral, Medtronic (dose unspecified) CB: Mustang, Boston Scientific	120 sec
Kitrou, EJR, 2015-AVF [9]	7	7	75	70	65.7 ± 13.2	62.5 ± 15.4	20	20	15	10	PCB: IN. PACT Admiral, Medtronic (3 µg/mm^2^) Control: HPB	Unspecified
Kitrou, JVIR, 2015 [10]	20	20	60	70	64.3 ± 14.5	57 ± 14.2	20	35	15	15	PCB: IN. PACT Admiral, Medtronic (3 µg/mm^2^) Control: HPB	90 sec
Lai, 2014 [11]	10	10	40		67.2 ± 9.4		50		40		PCB: SeQuent Please, B.Braun (dose unspecified) CB: unspecified	60 sec
Lookstein, 2020 [12]	170	160	65.9	63.1	65.8 ± 13.1	65.5 ± 13.4	62.9	68.8	91.2	94.4	PCB: IN. PACT Admiral, Medtronic (3.5 µg/mm^2^) Control: uncoated balloon	Unspecified
Maleux, 2018 [20]	33	31	72.7	58.1	69.3 ± 14.9	66.9 ± 17	–	–	–	–	PCB: IN. PACT Admiral, Invatec/Medtronic (dose unspecified) Control: Admiral Extreme, Invatec/Medtronic	Unspecified
Swinnen, 2018 [13]	68	60	61.8	61.7	65.2 ± 13.6	64.5 ± 13.9	55.9	65.0	–	–	PCB: IN. PACT Admiral/Pacific, Medtronic (3 µg/mm^2^) Control: uncoated angioplasty balloon of the operator’s choice	120 sec
Therasse, 2021-AVF [14]	60	60	83.3	83.3	63.5 ± 12.6	66.6 ± 12.6	61.7	71.7	86.7	81.7	PCB: Passeo-18 Lux, Biotronik (3 µg/mm^2^) CB: same type without drug	60 sec
Trerotola, 2020 [15]	141	144	61.7	59	64 ± 15	61 ± 13	58.2	65.3	94.3	98.6	PCB: Lutonix 035, Bard (2 µg/mm^2^) Control: control balloon of similar design but without drug coating	Unspecified
Yin, 2021 [16]	78	83	56.4	50.6	56 ± 13	54 ± 13	34.6	34.9	84.6	84.3	PCB: APERTO, Cardionovum (3 µg/mm^2^) Control: Ohicho II HPBs, Kaneka Corp	120–180 sec

PCB: paclitaxel coated balloon; CB: conventional balloon; sec: seconds; -: missing data.

### 3-Month TLPP

Six studies [8,11,15,17,20,21] evaluated the 3-month TLPP. The pooled rates for the PCB group and CB group were 86.8% (211/243) vs 85.5% (207/242), respectively. The meta-analysis showed that the difference of 3-month TLPP rates between two groups was not statistically significant (RR 1.00, 95% CI 0.93–1.07, *p* = 1.000, *I*^2^ = 33.5%, Cochrane *Q* test *p* = 0.185, fixed-effect model, Figure 4(A)). The funnel graph was roughly symmetrical which indicated the absence of significant publication bias (Egger’s test *p* = 0.563, Appendix 2).

Figure 4.Meta-analysis of TLPP at 3 months (A), 6 months (B), 9 months (C) and 12 months (D). TLPP: target lesion primary patency; PCB: paclitaxel coated balloon; CB: conventional balloon; CI: confidence interval.

**Figure:**
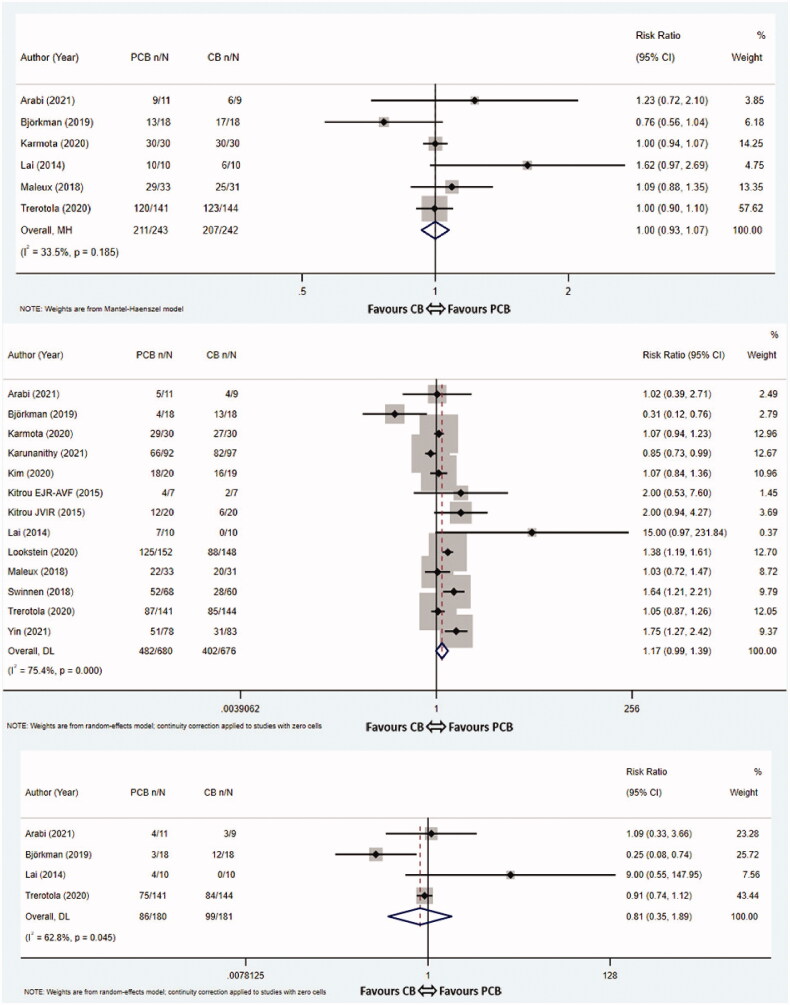

**Figure:**
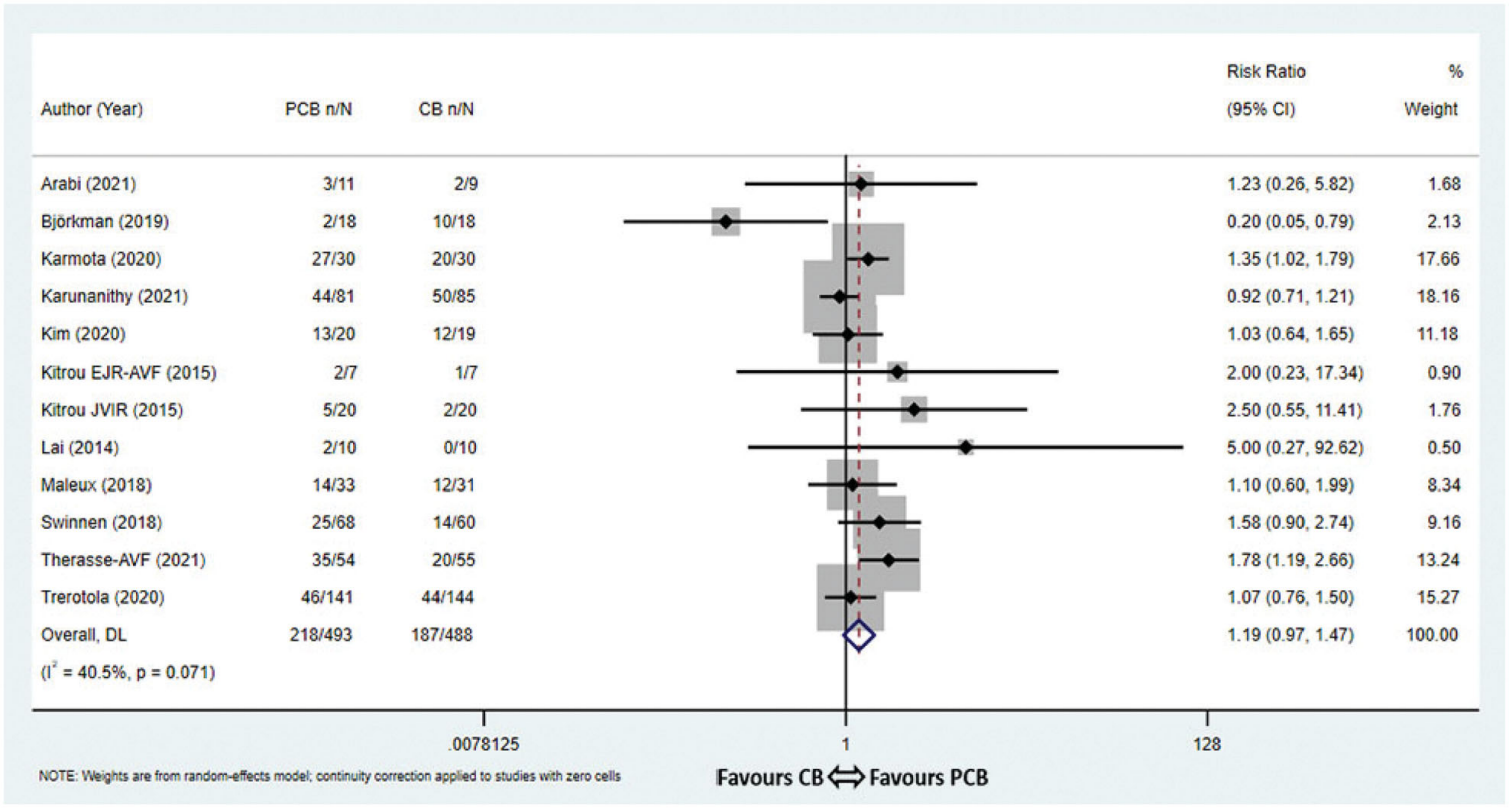

### 6-Month TLPP

Thirteen studies [8–13,15–21] investigated the 6-month TLPP. The pooled event rates at 6 months in the PCB group and the CB group were 70.9% (482/680) and 59.5% (402/676), respectively. While the PCB group did improve the 6-month TLPP rates, the difference did not reach statistical significance (RR 1.17, 95% CI 0.99–1.39, *p* = 0.065, *I*^2^ = 75.4%, Cochrane *Q* test *p* = 0.000, random-effect model, Figure 4(B)). In order to estimate the possible sources of heterogeneity, sensitivity analysis and subgroup analyses based on PCB type, paclitaxel dose and inflation time were performed for this outcome. It is a pity that subgroup analyses of study characteristics did not find any factors that accounted for the heterogeneity (Table 3). The results of a subgroup analysis according to PCB type indicated that the APERTO (Cardionovum) balloons significantly outperformed CBs in terms of 6-month TLPP (RR 1.75, 95% CI 1.27–2.42, *p* = 0.001, fixed-effect model), whereas the Lutonix 035 (Bard), IN.PACT Admiral (Medtronic) and SeQuent Please (B.Braun) balloons did not improve 6-month TLPP rates compared with CBs (RR 0.97, 95% CI 0.87–1.08, *p* = 0.557, *I*^2^ = 47.2%, Cochrane *Q* test *p* = 0.128, fixed-effect model; RR 1.22, 95% CI 0.96–1.57, *p* = 0.110, *I*^2^ = 67.1%, Cochrane *Q* test *p* = 0.006, random-effect model; RR 1.00, 95% CI 0.48–2.10, *p* = 1.000, fixed-effect model). A subgroup analysis stratified by paclitaxel dose revealed that treatment with standard-dose PCBs (3.0 μg/mm^2^ and 3.5 μg/mm^2^) were significantly effective than CBs at improving 6-month TLPP rates for dysfunctional AVFs (RR 1.43, 95% CI 1.07–1.91, *p* = 0.016, *I*^2^ = 65.2%, Cochrane *Q* test *p* = 0.013, random-effect model). The subgroup analysis based on inflation time showed that the dilation time of PCBs greater than 120 s did significantly improve 6-month TLPP rates compare with the controls (RR 1.29; 95% CI 1.03–1.63, *p* = 0.029, *I*^2^ = 68.9%, Cochrane *Q* test *p* = 0.012, random-effect model). We also did not find the source of heterogeneity through the sensitivity analysis. After removing one study, the estimates did not change significantly, which means these results were relatively robust in this meta-analysis (Appendix 2). The funnel graph was symmetrically distributed, and publication bias was not evident (Egger’s test *p* = 0.443, Appendix 2).

**Table 3.** Subgroup analyses.

Parameters	Factors	Subgroup	No. of trials	Effect estimate and 95% CI	*I*^2^ (%)	*P* value for *Q* statistic	*P* value	Effect model
6-month TLPP	PCB type	Lutonix 035, Bard	4	0.97 (0.87, 1.08)	47.2	0.128	0.557	Fixed-effect model
		IN.PACT Admiral, Medtronic	7	1.22 (0.96, 1.57)	67.1	0.006	0.110	Random-effect model
		SeQuent Please, B.Braun	1	1.00 (0.48, 2.10)	0.0	–	1.000	Fixed-effect model
		APERTO, Cardionovum	1	1.75 (1.27, 2.42)	0.0	–	0.001	Fixed-effect model
	Paclitaxel dose	Low-dose (2 μg/mm^2^)	3	0.95 (0.84, 1.08)	35.5	0.212	0.428	Fixed-effect model
		Standard-dose (3 μg/mm^2^ and 3.5 μg/mm^2^)	6	1.43 (1.07, 1.91)	65.2	0.013	0.016	Random-effect model
		Unspecified	4	1.00 (0.87, 1.15)	41.5	0.163	1.000	Fixed-effect model
	Inflation time	≥120 sec	5	1.29 (1.03, 1.63)	68.9	0.012	0.029	Random-effect model
		<120 sec	3	1.48 (0.26, 8.48)	85.0	0.001	0.662	Random-effect model
		Unspecified	5	1.08 (0.85, 1.38)	80.5	0.000	0.513	Random-effect model
12-month TLPP	PCB type	Lutonix 035, Bard	4	1.06 (0.88, 1.27)	23.9	0.268	0.535	Fixed-effect model
		IN.PACT Admiral, Medtronic	6	1.12 (0.83, 1.50)	44.5	0.109	0.467	Fixed-effect model
		Passeo-18 Lux, Biotronik	1	1.78 (1.19, 2.66)	0.0	–	0.005	Fixed-effect model
		SeQuent Please, B.Braun	1	1.00 (0.25, 4.00)	0.0	–	1.000	Fixed-effect model
	Paclitaxel dose	Low-dose (2 μg/mm^2^)	3	1.00 (0.80, 1.23)	0.0	0.764	0.978	Fixed-effect model
		Standard-dose (3 μg/mm^2^ and 3.5 μg/mm^2^)	5	1.33 (0.72, 2.48)	57.8	0.050	0.367	Random-effect model
		Unspecified	4	1.00 (0.80, 1.24)	47.3	0.127	1.000	Fixed-effect model
	Inflation time	≥120 sec	4	1.33 (1.04, 1.71)	0.0	0.676	0.023	Fixed-effect model
		<120 sec	4	1.24 (0.37, 4.09)	70.1	0.018	0.730	Random-effect model
		Unspecified	4	1.01 (0.83, 1.24)	0.0	0.801	0.898	Fixed-effect model

CI: confidence interval; TLPP: target lesion primary patency; PCB: paclitaxel coated balloon; CB: conventional balloon; sec: seconds.

### 9-Month TLPP

Data on 9-month TLPP were extracted from four articles [11,15,17,21]. The pooled rates for the PCB group and CB group were 47.8% (86/180) vs 54.7% (99/181), respectively. No statistically significant differences were observed between the two groups (RR 0.81, 95% CI 0.35–1.89, *p* = 0.625, *I*^2^ = 62.8%, Cochrane *Q* test *p* = 0.045, random-effect model, Figure 4(C)). There were too few RCTs to perform sensitivity analysis, subgroup analysis and publication bias test.

### 12-Month TLPP

Of twelve trials [8–11,13–15,17–21] reporting the 12-month TLPP or having sufficient data for extrapolation, the pooled 12-month TLPP rates were 44.2% (218/493) in the PCB group vs 40.4% (197/488) in the CB group, respectively. PCB angioplasty was not correlated with 12-month TLPP (RR 1.19, 95% CI 0.97–1.47, *p* = 0.096, *I*^2^ = 40.5%, Cochrane *Q* test *p* = 0.071, random-effect model, Figure 4(D)). Almost all of the subgroup analyses had no influence on the heterogeneity of the pooled analysis (Table 3). The outcome of subgroup analysis according to PCB types demonstrated that the Passeo-18 Lux (Biotronik) balloons were favored over CBs in terms of 12-month TLPP (RR 1.78, 95% CI 1.19–2.66, *p* = 0.005, fixed-effect model), whereas the Lutonix 035 (Bard), IN.PACT Admiral (Medtronic) and SeQuent Please (B.Braun) balloons did not improve 12-month TLPP compared with CBs (RR 1.06, 95% CI 0.88–1.27, *p* = 0.535, *I*^2^ = 23.9%, Cochrane *Q* test *p* = 0.268, fixed-effect model; RR 1.12, 95% CI 0.83–1.50, *p* = 0.467, *I*^2^ = 44.5%, Cochrane *Q* test *p* = 0.109, fixed-effect model; RR 1.00, 95% CI 0.25–4.00, *p* = 1.000, fixed-effect model). A subgroup analysis based on inflation time demonstrated that 12-month TLPP rates were significantly higher in the group with the PCB inflation time ≥120 s compared with the controls (RR 1.33, 95% CI 1.04–1.71, *p* = 0.023, *I*^2^ = 0.0%, Cochrane *Q* test *p* = 0.676, fixed-effect model). Additional sensitivity analysis was performed by eliminating each included study step by step, showing that the studies were reliable and robust (Appendix 2). No significant publication bias existed in the studies evaluating the 12-month TLPP rates (Egger’s test *p* = 0.740, Appendix 2).

### Technical success

**Figure 5.**
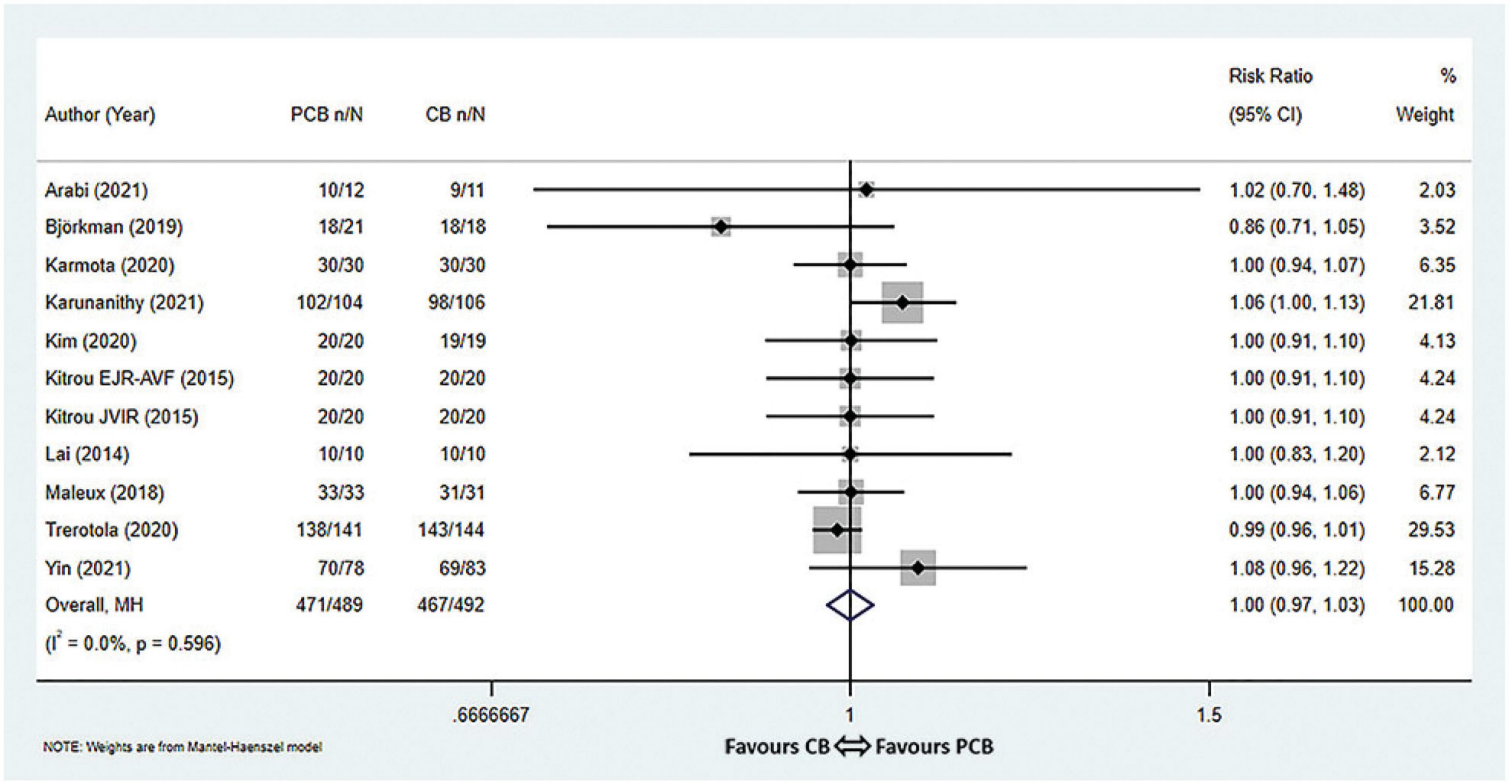
Meta-analysis of technical success rate. PCB: paclitaxel coated balloon; CB: conventional balloon; CI: confidence interval.

Altogether, eleven studies [8–11,15–21] reported the technical success rates in a total of 981 patients, with 489 assigned to the PCB group and 492 assigned to the CB group. The pooled rates for the PCB group and CB group were 96.3% (471/489) vs 94.9% (467/492), respectively. The results revealed that there was no statistically significant difference in technical success rates between the two groups (RR 1.00, 95% CI 0.97–1.03, *p* = 1.000, *I*^2^ = 0.0%, Cochrane *Q* test *p* = 0.596, fixed-effect model, Figure 5). The funnel plot was symmetrically distributed, indicating no remarkable publication bias in these studies (Egger’s test *p* = 0.751, Appendix 2).

### All-cause mortality

**Figure 6.**
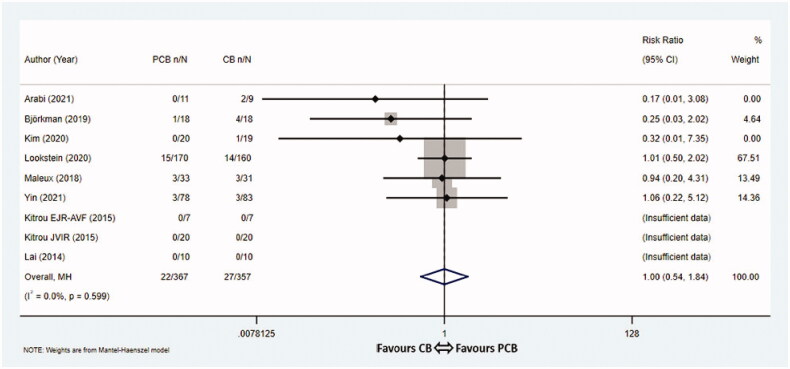
Meta-analysis of all-cause mortality. PCB: paclitaxel coated balloon; CB: conventional balloon; CI: confidence interval.

12-month mortality rates were documented in nine studies [9–12,16,17,19–21], among which three studies [9–11] documented zero death. These nine articles involved 724 patients, with 367 assigned to the PCB group and 357 assigned to the CB group. Overall, the pooled 12-month mortality rates were 6.0% (22/367) in the PCB group vs 7.6% (27/357) in the CB group, respectively. The statistical analysis showed no significant differences between the two groups with respect to 12-month mortality rates (RR 1.00, 95% CI 0.54–1.84, *p* = 1.000, *I*^2^ = 0.0%, Cochrane *Q* test *p* = 0.599, fixed-effect model, Figure 6). The associated funnel plot was basically symmetrical, suggesting no obvious publication bias (Egger’s test *p* = 0.055, Appendix 2).

## Discussion

In this meta-analysis, we conducted a comprehensive search for all studies regarding PCB angioplasty versus CB angioplasty in dysfunctional AVF. A total of fourteen articles containing 1535 subjects were included, and results showed no significant differences between the two groups in TLPP rates after 3, 6, 9 and 12 months of treatment (RR 1.00, 95% CI 0.93–1.07, *p* = 1.000, *I*^2^ = 33.5%; RR 1.17, 95% CI 0.99–1.39, *p* = 0.065, *I*^2^ = 75.4%; RR 0.81, 95% CI 0.35–1.89, *p* = 0.625, *I*^2^ = 62.8%; RR 1.19, 95% CI 0.97–1.47, *p* = 0.096, *I*^2^ = 40.5%). In addition, there were no significant differences observed in the technical success rates (RR 1.00, 95% CI 0.97–1.03, *p* = 1.000, *I*^2^ = 0%) and 12-month mortality rates (RR 1.00, 95% CI 0.54–1.84, *p* = 1.000, *I*^2^ = 0%) between the two groups. Moreover, a cost-effectiveness analysis conducted by Diehm *et al* revealed that the catheter material costs for PCBs and CBs were 2008 and 464 Swiss Francs per patient, respectively [40]. This is because while an uncoated balloon was used in the CB angioplasty, both an uncoated balloon for predilation and a PCB were required in the PCB angioplasty. From the above results, it appears that PCB angioplasty is neither more effective nor much safer and is more costly. Thereby, PCB angioplasty seems to be not cost-effective compared to CB angioplasty from an economic point of view.

Since the first RCT using PCB in AVF was conducted by Lai et al. [11], the debates on the benefits of PCB angioplasty for the treatment of dysfunctional AVF have never ceased. A number of meta-analyses evaluating the efficacy and safety of PCB angioplasty versus CB angioplasty for the treatment of dysfunctional AVF have been published [41–43]. In line with our findings, a recent meta-analysis by Liao *et al* demonstrated no significant improvement of TLPP rates in the PCB group, either for that at 6 months (RR 0.75, 95% CI 0.56–1.01, *p* = 0.06) or 12 months (RR 0.89, 95% CI 0.79–1.00, *p* = 0.06) [41]. Similar results have also been reported by Abdul Salim *et al* and Lazarides et al. [42,43]. In comparison to the meta-analyses described above, the strength of this paper lies in the incorporation of the most recent studies with large sample sizes into the final meta-analysis, making the results more persuasive.

Obvious heterogeneity existed in the included literature. We noticed that there was one study that obviously deviated from the axis of symmetry in the forest graph, which might have a great influence on heterogeneity [21]. Bjorkman *et al* suggested that the target lesion revascularization-free survival after the PCB angioplasty was clearly worse with 1-year follow-up, which was opposite to other studies [21]. In order to find out the source of heterogeneity, sensitivity analyses were performed by omitting one study at a time. The results of sensitivity analyses showed that there were no significant changes in the overall effect measures, indicating the results were relatively reliable.

In extension, we performed subgroup analyses based on paclitaxel dose, PCB type and inflation time. Of interest, the 6-month outcomes of the endovascular invention utilizing PCBs for dysfunctional AVFs were linked with the doses of paclitaxel delivered to vessels. We found that standard-dose PCBs (3.0 μg/mm^2^ and 3.5 μg/mm^2^) were significantly more effective compared with CBs in improving 6-month TLPP rates, while there were no significant differences between low-dose PCBs (2.0 μg/mm^2^) and CBs at 6-month TLPP rates. Katsanos and colleagues, pooling data from eleven RCTs, showed that standard-dose PCBs (3.0 μg/mm^2^ and 3.5 μg/mm^2^) were superior to low-dose PCBs (2.0 μg/mm^2^) in reducing the rates of restenosis and target lesion revascularization (TLR) in the femoropopliteal artery, a finding that was generally consistent with the conclusions of our meta-analysis [44]. For these, the current study recommended that priority should be given to standard-dose PCBs (3.0 μg/mm^2^ and 3.5 μg/mm^2^) in the treatment of dysfunctional AVFs.

Meanwhile, we also found that APERTO (Cardionovum) balloons significantly outperformed CBs in terms of 6-month TLPP, whereas the Lutonix 035 (Bard), IN.PACT Admiral (Medtronic) and SeQuent Please (B.Braun) balloons did not improve 6-month TLPP rates compared with CBs. And Passeo-18 Lux (Biotronik) balloons were favored over CBs in terms of 12-month TLPP, whereas the Lutonix 035 (Bard), IN.PACT Admiral (Medtronic) and SeQuent Please (B.Braun) balloons did not improve 12-month TLPP rates compared with CBs. We speculated that this may be explained by the different doses of drug delivered to vessels.

Another interesting finding was that 6- and 12-month TLPP rates were significantly higher in the group with the PCB inflation time ≥120 s compared with the controls. However, no significant differences were detected between the group with the PCB inflation time <120 s and the control group. In agreement with this study, Rhee and colleagues also reported that fully optimized PCB angioplasty with prolonged inflation time plays an important role in reducing target lesion failure after PCB angioplasty [45].

Although all of the enrolled studies were RCTs, allowing our findings to be reliable, several limitations should be acknowledged. Firstly, because of the macroscopic differences between PCBs and CBs, investigators cannot be unaware of the treatment assignment. None of the included RCTs was double-blinded, ineluctably increasing the risk of bias. Secondly, heterogeneity existed in the included literature. Sensitivity analyses and subgroup analyses according to PCB type, paclitaxel dose and inflation time were conducted to find the source of heterogeneity, compensating for this deficiency to some extent. However, the subgroup analysis based on AVF age was not performed in this meta-analysis. TLPP has been shown to have a positive correlation with AVF age in a previous study by Irani *et al* [28]. Thirdly, not all included articles reported the six outcomes and the data used for meta-analysis were incomplete, despite our efforts to contact authors of the included studies. Fourthly, all follow-ups were clinically driven. That is to say, not all patients have undergone ultrasound examinations before endovascular interventions, therefore causing a potential bias. Fifthly, unpublished results were not available, which inevitably produced publication bias. Finally, it should be acknowledged that the present study was not registered, with the possibility of a small offset. But it has to be pointed out that our meta-analysis was conducted strictly in compliance with the process of a systematic review.

In conclusion, this meta-analysis showed that there was insufficient evidence to support the distinct superiority of PCB angioplasty over CB angioplasty in the treatment of dysfunctional AVF. Due to the heterogeneity across studies, the findings from our study should be dealt with with some caution, although sensitivity analyses and subgroup analyses were performed to compensate for this deficiency to some extent. Thus, more multi-center, large-scale and well-designed RCTs are required to confirm our conclusions in the future.

## Figures and Tables

**Figure A1.**
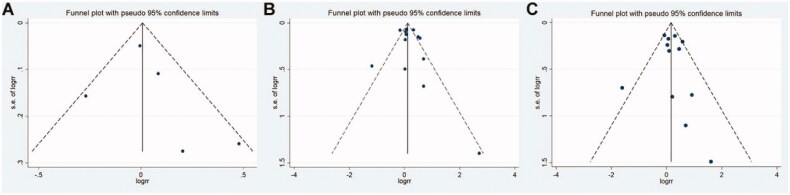
Funnel plot of TLPP at 3 months (A), 6 months (B) and 12 months (C). TLPP: target lesion primary patency.

**Figure A2.**
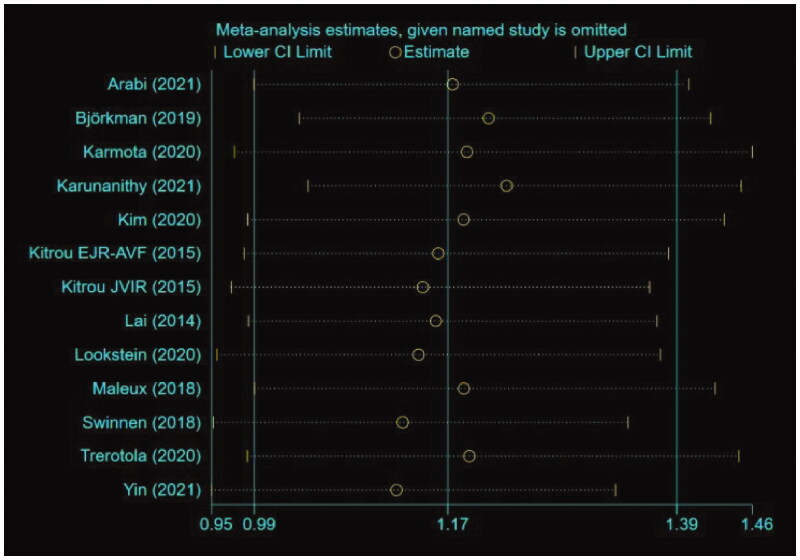
Sensitivity analysis of TLPP at 6 months. TLPP: target lesion primary patency; CI: confidence interval.

**Figure A3.**
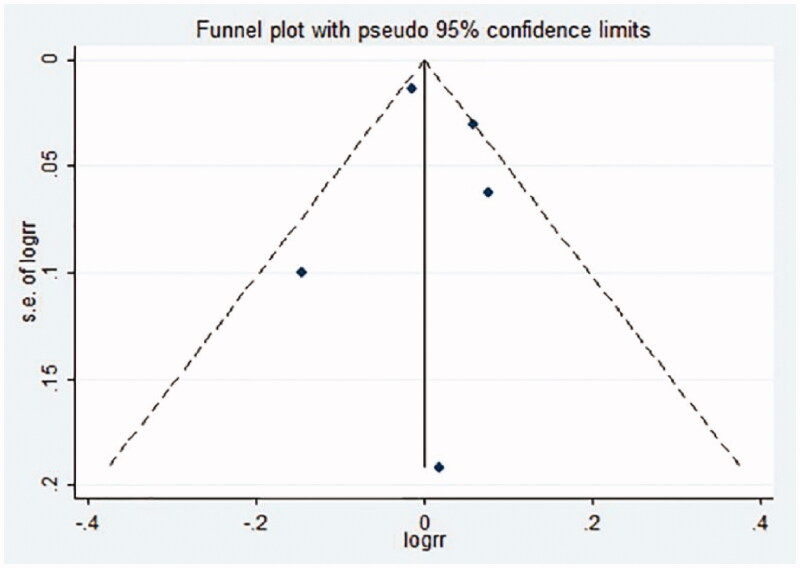
Funnel plot of technical success rate.

**Figure A4.**
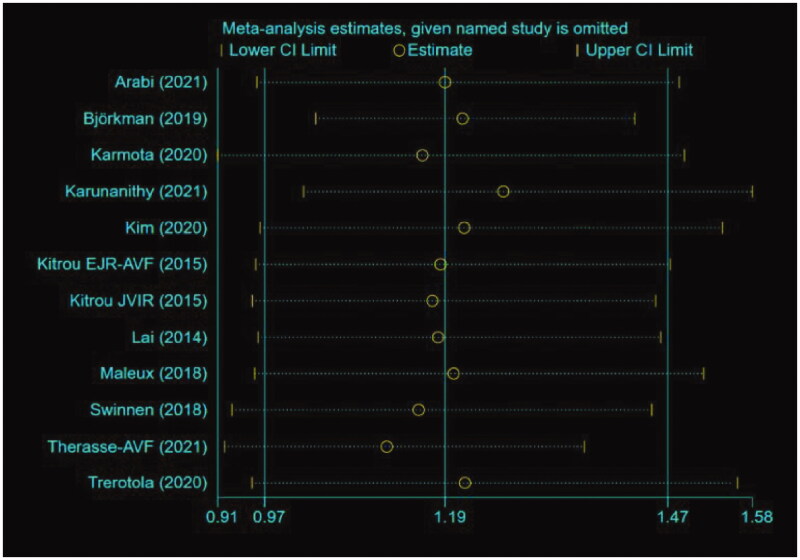
Sensitivity analysis of TLPP at 12 months. TLPP: target lesion primary patency; CI: confidence interval.

## Data Availability

Not applicable.

## Appendix 1. The table that illustrates search terms

**Table:**

PubMed	
((((((((("Arteriovenous Fistula"[Mesh]) OR (((((((((((((((((((((fistula, arteriovenous[Title/Abstract]) OR (fistulas, arteriovenous[Title/Abstract])) OR (aneurysm, arteriovenous[Title/Abstract])) OR (anastomosis arteriovenosa[Title/Abstract])) OR (arterial venous anastomosis[Title/Abstract])) OR (arterio venous anastomosis[Title/Abstract])) OR (arterio venous aneurysm[Title/Abstract])) OR (arterio-venous fistula[Title/Abstract])) OR (arterio-venous fistulae[Title/Abstract])) OR (arterio-venous fistulas[Title/Abstract])) OR (arteriovenous anastomosis[Title/Abstract])) OR (arteriovenous aneurysm[Title/Abstract])) OR (arteriovenous crossing[Title/Abstract])) OR (arteriovenous fistulas[Title/Abstract])) OR (arteriovenous fistulae[Title/Abstract])) OR (artery vein fistula[Title/Abstract])) OR (av anastomosis[Title/Abstract])) OR (av aneurysm[Title/Abstract])) OR (AV fistula[Title/Abstract])) OR (AV fistulae[Title/Abstract])) OR (AV fistulas[Title/Abstract]))) OR (arteriovenous access[Title/Abstract])) OR (hemodialysis fistulas[Title/Abstract])) OR (hemodialysis access[Title/Abstract])) OR (dialysis fistulas[Title/Abstract])) OR (dialysis access[Title/Abstract])) OR (dialysis fistula[Title/Abstract])) OR (dialysis fistulae[Title/Abstract])) AND ((("Paclitaxel"[Mesh]) OR (((((((((((((((((((((((((((((((((((((((((((((((((((((((((((7-epi-taxol[Title/Abstract]) OR (7 epi taxol[Title/Abstract]))) OR (abi 007[Title/Abstract])) OR (abi007[Title/Abstract])) OR (abraxane[Title/Abstract])) OR (albumin bound paclitaxel[Title/Abstract])) OR (albumin-bound paclitaxel[Title/Abstract])) OR (anzatax[Title/Abstract])) OR (apealea[Title/Abstract])) OR (asotax[Title/Abstract])) OR (biotax[Title/Abstract])) OR (bms 181339[Title/Abstract])) OR (bms181339[Title/Abstract])) OR (bmy 45622[Title/Abstract])) OR (bmy45622[Title/Abstract])) OR (bris taxol[Title/Abstract])) OR (bristaxol[Title/Abstract])) OR (britaxol[Title/Abstract])) OR (coroxane[Title/Abstract])) OR (dts 301[Title/Abstract])) OR (dts301[Title/Abstract])) OR (endotag-1[Title/Abstract])) OR (formoxol[Title/Abstract])) OR (genexol[Title/Abstract])) OR (genexol pm[Title/Abstract])) OR (hunxol[Title/Abstract])) OR (ifaxol[Title/Abstract])) OR (infinnium[Title/Abstract])) OR (intaxel[Title/Abstract])) OR (mbt 0206[Title/Abstract])) OR (mbt0206[Title/Abstract])) OR (medixel[Title/Abstract])) OR (mitotax[Title/Abstract])) OR (nab paclitaxel[Title/Abstract])) OR (nanoparticle albumin bound paclitaxel[Title/Abstract])) OR (nsc-125973[Title/Abstract])) OR (nsc 125973[Title/Abstract])) OR (nsc 673089[Title/Abstract])) OR (nsc125973[Title/Abstract])) OR (nsc673089[Title/Abstract])) OR (oas pac 100[Title/Abstract])) OR (oaspac100[Title/Abstract])) OR (oncogel[Title/Abstract])) OR (onxol[Title/Abstract])) OR (paclitaxel, (4 alpha)-Isomer[Title/Abstract])) OR (pacitaxel[Title/Abstract])) OR (paclitaxel nab[Title/Abstract])) OR (pacxel[Title/Abstract])) OR (padexol[Title/Abstract])) OR (parexel[Title/Abstract])) OR (paxceed[Title/Abstract])) OR (paxene[Title/Abstract])) OR (paxus[Title/Abstract])) OR (pazenir[Title/Abstract])) OR (praxel[Title/Abstract])) OR (sb 05 (terpenoid)[Title/Abstract])) OR (sb05 (terpenoid)[Title/Abstract])) OR (taxocris[Title/Abstract]))) OR ((((((((((((drug eluting balloons[Title/Abstract]) OR (drug-eluting balloon[Title/Abstract])) OR (balloon, drug-eluting[Title/Abstract])) OR (balloons, drug-eluting[Title/Abstract])) OR (balloons, drug eluting[Title/Abstract])) OR (drug-coated balloons[Title/Abstract])) OR (drug coated balloons[Title/Abstract])) OR (drug-coated balloon[Title/Abstract])) OR (balloon, drug-coated[Title/Abstract])) OR (balloons, drug-coated[Title/Abstract])) OR (balloons, drug coated[Title/Abstract])) OR (Passeo-18 Lux[Title/Abstract])))	
**Web of Science**	
#1	TS=(Arteriovenous Fistula OR fistula, arteriovenous OR fistulas, arteriovenous OR aneurysm, arteriovenous OR anastomosis arteriovenosa OR arterial venous anastomosis OR arterio venous anastomosis OR arterio venous aneurysm OR arterio-venous fistula OR arterio-venous fistulae OR arterio-venous fistulas OR arteriovenous anastomosis OR arteriovenous aneurysm OR arteriovenous crossing OR arteriovenous fistulas OR arteriovenous fistulae OR artery vein fistula OR av anastomosis OR av aneurysm OR AV fistula OR AV fistulae OR AV fistulas OR arteriovenous access OR hemodialysis fistulas OR hemodialysis access OR dialysis fistulas OR dialysis access OR dialysis fistula OR dialysis fistulae)
#2	TS=(drug eluting balloons OR drug-eluting balloon OR balloon, drug-eluting OR balloons, drug-eluting OR balloons, drug eluting OR drug-coated balloons OR drug coated balloons OR drug-coated balloon OR balloon, drug-coated OR balloons, drug-coated OR balloons, drug coated OR Passeo-18 Lux)
#3	TS=(Paclitaxel OR 7-epi-taxol OR 7 epi taxol OR abi 007 OR abi007 OR abraxane OR albumin bound paclitaxel OR albumin-bound paclitaxel OR anzatax OR apealea OR asotax OR biotax OR bms 181339 OR bms181339 OR bmy 45622 OR bmy45622 OR bris taxol OR bristaxol OR britaxol OR coroxane OR dts 301 OR dts301 OR endotag-1 OR formoxol OR genexol OR genexol pm OR hunxol OR ifaxol OR infinnium OR intaxel OR mbt 0206 OR mbt0206 OR medixel OR mitotax OR nab paclitaxel OR nanoparticle albumin bound paclitaxel OR nsc-125973 OR nsc 125973 OR nsc 673089 OR nsc125973 OR nsc673089 OR oas pac 100 OR oaspac100 OR oncogel OR onxol OR paclitaxel, (4 alpha)-Isomer OR pacitaxel OR paclitaxel nab OR pacxel OR padexol OR parexel OR paxceed OR paxene OR paxus OR pazenir OR praxel OR sb 05 (terpenoid) OR sb05 (terpenoid) OR taxocris OR taxol OR taxol A OR taxol, bris OR taxus (drug) OR taycovit OR yewtaxan)
#4	#2 OR #3
#5	#1 AND #4
**Embase**	
#1	'arteriovenous fistula'/exp
#2	'fistula, arteriovenous':ab,ti OR 'fistulas, arteriovenous':ab,ti OR 'aneurysm, arteriovenous':ab,ti OR 'anastomosis arteriovenosa':ab,ti OR 'arterial venous anastomosis':ab,ti OR 'arterio venous anastomosis':ab,ti OR 'arterio venous aneurysm':ab,ti OR 'arterio-venous fistula':ab,ti OR 'arterio-venous fistulae':ab,ti OR 'arterio-venous fistulas':ab,ti OR 'arteriovenous anastomosis':ab,ti OR 'arteriovenous aneurysm':ab,ti OR 'arteriovenous crossing':ab,ti OR 'arteriovenous fistulas':ab,ti OR 'arteriovenous fistulae':ab,ti OR 'artery vein fistula':ab,ti OR 'av anastomosis':ab,ti OR 'av aneurysm':ab,ti OR 'av fistula':ab,ti OR 'av fistulae':ab,ti OR 'av fistulas':ab,ti OR 'arteriovenous access':ab,ti OR 'hemodialysis fistulas':ab,ti OR 'hemodialysis access':ab,ti OR 'dialysis fistulas':ab,ti OR 'dialysis access':ab,ti OR 'dialysis fistula':ab,ti OR 'dialysis fistulae':ab,ti
#3	#1 OR #2
#4	'drug-coated balloon'/exp
#5	'drug eluting balloons':ab,ti OR 'drug-eluting balloon':ab,ti OR 'balloon, drug-eluting':ab,ti OR 'balloons, drug-eluting':ab,ti OR 'balloons, drug eluting':ab,ti OR 'drug-coated balloons':ab,ti OR 'drug coated balloons':ab,ti OR 'balloon, drug-coated':ab,ti OR 'balloons, drug-coated':ab,ti OR 'balloons, drug coated':ab,ti OR 'passeo-18 lux':ab,ti
#6	#4 OR #5
#7	'paclitaxel'/exp
#8	'7-epi-taxol':ab,ti OR '7 epi taxol':ab,ti OR 'abi 007':ab,ti OR 'abi007':ab,ti OR 'abraxane':ab,ti OR 'albumin bound paclitaxel':ab,ti OR 'albumin-bound paclitaxel':ab,ti OR 'anzatax':ab,ti OR 'apealea':ab,ti OR 'asotax':ab,ti OR 'biotax':ab,ti OR 'bms 181339':ab,ti OR 'bms181339':ab,ti OR 'bmy 45622':ab,ti OR 'bmy45622':ab,ti OR 'bris taxol':ab,ti OR 'bristaxol':ab,ti OR 'britaxol':ab,ti OR 'coroxane':ab,ti OR 'dts 301':ab,ti OR 'dts301':ab,ti OR 'endotag-1′:ab,ti OR 'formoxol':ab,ti OR 'genexol':ab,ti OR 'genexol pm':ab,ti OR 'hunxol':ab,ti OR 'ifaxol':ab,ti OR 'infinnium':ab,ti OR 'intaxel':ab,ti OR 'mbt 0206':ab,ti OR 'mbt0206':ab,ti OR 'medixel':ab,ti OR 'mitotax':ab,ti OR 'nab paclitaxel':ab,ti OR 'nanoparticle albumin bound paclitaxel':ab,ti OR 'nsc-125973':ab,ti OR 'nsc 125973':ab,ti OR 'nsc 673089':ab,ti OR 'nsc125973':ab,ti OR 'nsc673089':ab,ti OR 'oas pac 100':ab,ti OR 'oaspac100':ab,ti OR 'oncogel':ab,ti OR 'onxol':ab,ti OR 'paclitaxel, (4 alpha)-isomer':ab,ti OR 'pacitaxel':ab,ti OR 'paclitaxel nab':ab,ti OR 'pacxel':ab,ti OR 'padexol':ab,ti OR 'parexel':ab,ti OR 'paxceed':ab,ti OR 'paxene':ab,ti OR 'paxus':ab,ti OR 'pazenir':ab,ti OR 'praxel':ab,ti OR 'sb 05 (terpenoid)':ab,ti OR 'sb05 (terpenoid)':ab,ti OR 'taxocris':ab,ti OR 'taxol':ab,ti OR 'taxol a':ab,ti OR 'taxol, bris':ab,ti OR 'taxus (drug)':ab,ti OR 'taycovit':ab,ti OR 'yewtaxan':ab,ti
#9	#7 OR #8
#10	#6 OR #9
#11	#3 AND #10
**Cochrane Library**	
#1	(Arteriovenous Fistula):ti,ab,kw OR (fistula, arteriovenous):ti,ab,kw OR (fistulas, arteriovenous):ti,ab,kw OR (aneurysm, arteriovenous):ti,ab,kw OR (anastomosis arteriovenosa):ti,ab,kw OR (arterial venous anastomosis):ti,ab,kw OR (arterio venous anastomosis):ti,ab,kw OR (arterio venous aneurysm):ti,ab,kw OR (arterio-venous fistula):ti,ab,kw OR (arterio-venous fistulae):ti,ab,kw OR (arterio-venous fistulas):ti,ab,kw OR (arteriovenous anastomosis):ti,ab,kw OR (arteriovenous aneurysm):ti,ab,kw OR (arteriovenous crossing):ti,ab,kw OR (arteriovenous fistulas):ti,ab,kw OR (arteriovenous fistulae):ti,ab,kw OR (artery vein fistula):ti,ab,kw OR (av anastomosis):ti,ab,kw OR (av aneurysm):ti,ab,kw OR (AV fistula):ti,ab,kw OR (AV fistulae):ti,ab,kw OR (AV fistulas):ti,ab,kw OR (arteriovenous access):ti,ab,kw OR (hemodialysis fistulas):ti,ab,kw OR (hemodialysis access):ti,ab,kw OR (dialysis fistulas):ti,ab,kw OR (dialysis access):ti,ab,kw OR (dialysis fistula):ti,ab,kw OR (dialysis fistulae):ti,ab,kw
#2	(drug eluting balloons):ti,ab,kw OR (drug-eluting balloon):ti,ab,kw OR (balloon, drug-eluting):ti,ab,kw OR (balloons, drug-eluting):ti,ab,kw OR (balloons, drug eluting):ti,ab,kw OR (drug-coated balloons):ti,ab,kw OR (drug coated balloons):ti,ab,kw OR (drug-coated balloon):ti,ab,kw OR (balloon, drug-coated):ti,ab,kw OR (balloons, drug-coated):ti,ab,kw OR (balloons, drug coated):ti,ab,kw OR (Passeo-18 Lux):ti,ab,kw
#3	(Paclitaxel):ti,ab,kw OR (7 epi taxol):ti,ab,kw OR (abi 007):ti,ab,kw OR (abi007):ti,ab,kw OR (abraxane):ti,ab,kw OR (albumin bound paclitaxel):ti,ab,kw OR (albumin-bound paclitaxel):ti,ab,kw OR (anzatax):ti,ab,kw OR (apealea):ti,ab,kw OR (asotax):ti,ab,kw OR (biotax):ti,ab,kw OR (bms 181339):ti,ab,kw OR (bms181339):ti,ab,kw OR (bmy 45622):ti,ab,kw OR (bmy45622):ti,ab,kw OR (bris taxol):ti,ab,kw OR (bristaxol):ti,ab,kw OR (britaxol):ti,ab,kw OR (coroxane):ti,ab,kw OR (dts 301):ti,ab,kw OR (dts301):ti,ab,kw OR (endotag-1):ti,ab,kw OR (formoxol):ti,ab,kw OR (genexol):ti,ab,kw OR (genexol pm):ti,ab,kw OR (hunxol):ti,ab,kw OR (ifaxol):ti,ab,kw OR (infinnium):ti,ab,kw OR (intaxel):ti,ab,kw OR (mbt 0206):ti,ab,kw OR (mbt0206):ti,ab,kw OR (medixel):ti,ab,kw OR (mitotax):ti,ab,kw OR (nab paclitaxel):ti,ab,kw OR (nanoparticle albumin bound paclitaxel):ti,ab,kw OR (nsc-125973):ti,ab,kw OR (nsc 125973):ti,ab,kw OR (nsc 673089):ti,ab,kw OR (nsc125973):ti,ab,kw OR (nsc673089):ti,ab,kw OR (oas pac 100):ti,ab,kw OR (oaspac100):ti,ab,kw OR (oncogel):ti,ab,kw OR (onxol):ti,ab,kw OR (pacitaxel):ti,ab,kw OR (paclitaxel nab):ti,ab,kw OR (pacxel):ti,ab,kw OR (padexol):ti,ab,kw OR (parexel):ti,ab,kw OR (paxceed):ti,ab,kw OR (paxene):ti,ab,kw OR (paxus):ti,ab,kw OR (pazenir):ti,ab,kw OR (praxel):ti,ab,kw OR (taxocris):ti,ab,kw OR (taxol):ti,ab,kw OR (taxol A):ti,ab,kw OR (taxol, bris):ti,ab,kw OR (taycovit):ti,ab,kw OR (yewtaxan):ti,ab,kw
#4	#2 OR #3
#5	#1 AND #4
